# *Rosa canina* L. Methanol Extract and Its Component Rutin Reduce Cholesterol More Efficiently than Miglustat in Niemann–Pick C Fibroblasts

**DOI:** 10.3390/ijms252111361

**Published:** 2024-10-22

**Authors:** Dalanda Wanes, Sherin Al Aoua, Hadeel Shammas, Friederike Walters, Anibh M. Das, Sandra Rizk, Hassan Y. Naim

**Affiliations:** 1 Department of Biochemistry, University of Veterinary Medicine Hannover, Bünteweg 17, 30559 Hannover, Germany; wanes.dalanda@gmail.com (D.W.); alaoua.sherin@mh-hannover.de (S.A.A.); hshammas@zevra.com (H.S.); friederike.walters@tiho-hannover.de (F.W.); 2 Department of Paediatrics, Hannover Medical School, Carl-Neuberg-Strasse 1, 30625 Hannover, Germany; das.anibh@mh-hannover.de; 3 Department of Natural Sciences, Lebanese American University, Byblos P.O. Box 36, Lebanon; sandra.rizk@lau.edu.lb

**Keywords:** Niemann–Pick type C, *Rosa canina* L. methanol extract, miglustat (*N*-butyldeoxynojirimycin), trafficking, cholesterol, rutin, quercitrin

## Abstract

Niemann–Pick type C (NPC) disease is an autosomal recessive lysosomal storage disorder where 95% of the cases are caused by mutations in the Niemann–Pick C1 (NPC1) gene. Loss of function in NPC1 mutants trigger the accumulation of cholesterol in late endo-lysosomes and lysosomal dysfunction. The current study examined the potential of polyphenol-rich methanol extracts from *Rosa canina* L. (RCME) and two of its components, rutin and quercitrin, to enhance protein trafficking of NPC1 and restore cholesterol levels in fibroblasts derived from NPC patients, in comparison with miglustat, a drug approved in Europe for NPC treatment. Interestingly, RCME improved the trafficking of the compound heterozygous mutant NPC1^I1061T/P887L^, homozygous mutant NPC1^R1266Q^, and heterozygous mutant NPC1^N1156S^ between the endoplasmic reticulum and the Golgi and significantly reduced the levels of cellular cholesterol in the cell lines examined. Miglustat did not affect the trafficking of the three NPC1 mutants individually nor in combination with RCME. Markedly, rutin and quercitrin exerted their effects on cholesterol, but not in the trafficking pathway of NPC1, indicating that other components in RCME are implicated in regulating the trafficking of NPC1 mutants. By virtue of its dual function in targeting the trafficking of mutants of NPC1 as well as the cholesterol contents, RCME is more beneficial than available drugs that target substrate reduction and should be therefore considered in further studies for its feasibility as a therapeutic agent for NPC patients.

## 1. Introduction

Niemann–Pick disease type C (NPC) is a lysosomal storage disease that is characterized by progressive neurological deterioration due to mutations in the genes encoding the NPC1 (95% of cases) or NPC2 (5% of cases) proteins. It is inherited in an autosomal recessive manner and has an estimated incidence of 1/92,104 live births [1,2]. NPC1 and NPC2 are implicated in intracellular lipid transport, specifically the export of cholesterol from the lysosomes [3], and their malfunctioning triggers an excessive accumulation of unesterified cholesterol in lysosomes of numerous cell types, eventually leading to severe heterogeneous clinical manifestations, including neurological, visceral, or psychiatric symptoms and ultimately to death. In addition to cholesterol, glycosphingolipids, diacylglycerol, phospholipids, sterols, and ceramides accumulate in NPC cells [4].

NPC1 is a transmembrane glycoprotein comprising 1278 amino acids, spans the membrane 13 times, is synthesized and processed in the endoplasmic reticulum (ER), and finally is targeted along the secretory pathway to the lysosomes. Among the distinct protein domains of NPC1 are the N-terminal cholesterol luminal binding domain, a cysteine-rich luminal loop, and a cytosolic sterol-sensing domain [5]. The impairment of the intracellular cholesterol transport in NPC cells and its accumulation in the lysosomes leads to an overall elevation of cholesterol [6], since the de novo cholesterol synthesis in the ER and low-density lipoprotein (LDL) receptor synthesis remain unaffected. The continuous accumulation of cholesterol promotes the lysosomes into storage-like organelles. Since NPC cells cannot utilize exogenous LDL-derived cholesterol, they are largely dependent on the de novo cholesterol synthesis for cellular functions that require cholesterol [7]. For this reason, NPC appears to be a paradoxical disease showing mutual signs of storage and deficiency. Today, there is no curative therapy available for NPC. Miglustat is the only available disease-specific compound, which was approved in the European Union in 2009 for pediatric and adult NPC patients. Miglustat reduces substrate accumulations, especially in the brain, and aids in combating the neurological symptoms of NPC. Unfortunately, miglustat causes common side effects such as carbohydrate malabsorption, which stems from the inhibitory effect of miglustat on the intestinal disaccharidases, sucrase-isomaltase, and maltase-glucoamylase [8].

Cyclodextrin, another drug that was approved by the FDA, is capable of sequestering stored cholesterol in the late endosomes and lysosomes [9]. One of the main disadvantages of this drug is its inability to cross the blood–brain barrier.

Therapies with these drugs show variable improvements in the clinical course of the patients, which stem from different disease patterns of NPC. The variations in the clinical symptoms and response to therapies can be explained by heterogeneities in the trafficking patterns of NPC1 mutants [10] that correlate with variations in lipid storage, membrane composition, and miglustat amenability [11]. These observations favor patient’s personalized therapies that combine more than one drug with different targets.

Recently, we demonstrated that a methanol extract of *Rosa canina* L. (RCME), which is enriched in natural polyphenols and exhibits antioxidative in addition to anti-inflammatory properties, positively impacts membrane integrity and protein trafficking in dextran sulfate sodium (DSS)-treated colon carcinoma Caco-2 cells [12]. Particularly, abnormalities in cholesterol- and sphingolipid-enriched lipid rafts can be restored by RCME. These findings were linked to the effects of RCME within the ER milieu and in the Golgi apparatus.

*Rosa canina* L. belongs to the *Rosaceae* family and is distributed in temperate and subtropical zones of the northern hemisphere [13]. It is rich in natural polyphenols, has antioxidative and anti-inflammatory properties, and is traditionally used as a medicinal plant. A liquid chromatography–electrospray ionization–tandem mass spectrometry of the RCME used in this study was performed by Wanes et al. [14], identifying a total of 15 phenolic compounds, of which 7 were phenolic acids and 8 flavonoids. The flavonoids quercitrin and rutin, as well as the phenolic acids gallic acid and quinic acid, constitute the principal components of RCME.

Over 400 mutations were identified in the gene coding NPC1, with substantial variability in their prevalence across different populations [15,16]. The I1061T mutation, for example, is the most common variant in European populations and leads to misfolding of the NPC1 protein in the ER, while other mutations, such as R1266Q, result in different trafficking defects [17]. Such variations in NPC1 mutations pose challenges to developing broad therapeutic strategies, as their efficacy may vary based on the underlying genetic mutation [10,18]. The methanolic extract of *Rosa canina* demonstrated potential in modulating cholesterol levels and improving protein trafficking in the cell lines examined. Nevertheless, further analyses with many more cell lines from NPC patients are required to assess its general therapeutic applicability in NPC.

The aim of this study was to assess the ability of RCME to improve the trafficking of NPC1 and to reduce cholesterol levels in fibroblasts derived from four NPC patients’ skin biopsies harboring different NPC1 mutations.

## 2. Results

### 2.1. Variations in the Trafficking Behavior, Expression, and Cholesterol Content of NPC1 Mutants

**Figure 1:**
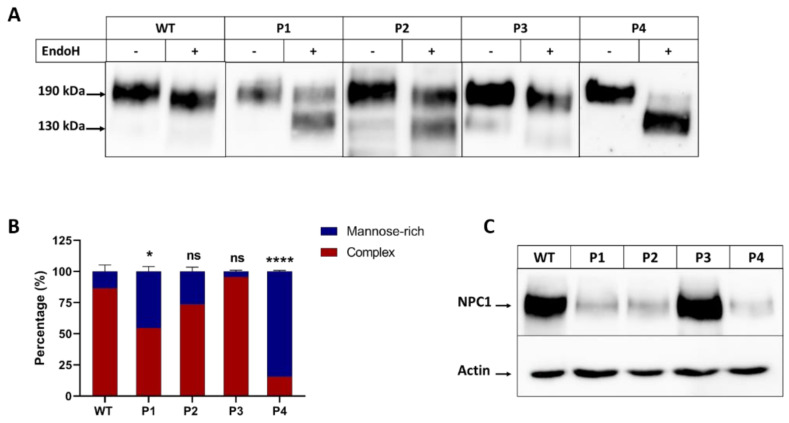
Biosynthetic forms of NPC1 proteins in different patients. The cellular lysates from fibroblasts derived from a healthy donor (WT), patient 1 (P1), patient 2 (P2), patient 3 (P3), and patient 4 (P4) were immunoprecipitated with anti-NPC1 and further treated or not treated with endo H. The mature complex glycosylated endo H-resistant form appears as a 190 kDa band and the endo H-sensitive immature form is cleaved to a 130 kDa band. The immunoprecipitates were subjected to SDS-PAGE and Western blot for NPC1 analysis (**A**). The band intensities of the complex and mannose-rich forms of the NPC1 protein were quantified and presented as percentage of the total (**B**). Equal protein amounts of cellular lysates were subjected to SDS-PAGE for NPC1 expression analysis versus the house keeping protein, actin (**C**). Ordinary one-way ANOVA, ns *p* > 0.05, * *p* < 0.05, and **** *p* < 0.0005 versus WT, S.E.M., and *n* = 3.

To identify the biosynthetic forms of NPC1 mutants (NPC1^MUT^) in comparison to NPC1 wild-type (NPC1^WT^), the immunoprecipitates were treated with endo H. Endo H cleaves the mannose-rich immature forms of proteins in the ER and early stages of the secretory pathway, while the complex glycosylated mature form of glycoproteins that were processed in the Golgi apparatus are endo H-resistant. The NPC1^WT^ was predominantly resistant to endo H, indicating that the major proportion of NPC1^WT^ at a steady state (~190 kDa protein) was trafficked along the secretory pathway to the complex glycosylated form (>85%) (Figure 1A,B). The NPC1 in the different patients’ fibroblasts exhibited variations in their protein forms upon treatments with endo H indicative of heterogeneity in their trafficking patterns between the ER and the Golgi. In patient 4 (P4), who is homozygous for the mutation p.R1266Q, NPC1^R1266Q^ was predominantly blocked in the ER, as shown by its entire sensitivity to endo H and a shift to a 130 kDa protein band. On the other hand, NPC1^I1061T/P887L^ in patient 1 (P1) (compound heterozygous to p.I1061T and p.P887L) and NPC1^N1156S^ in patient 2 (P2) (heterozygous for p.N1156S), revealed delayed trafficking patterns between the ER and the Golgi, as reflected by the proportions of the endo H-sensitive band (~50% and 26%, respectively). Notably, in patient 3 (P3), who is compound heterozygous for p.V1165M/p.0 mutations, NPC1^V1165M^ showed a biosynthetic pattern similar to NPC^WT^. Furthermore, the expression levels of the mutants at a steady state versus actin as a housekeeping protein varied. In fact, NPC1^I1061T/P887L^, NPC1^N1156S^, and NPC1^R1266Q^ in patients P1, P2, and P4, respectively, showed a reduced expression level concomitant with substantial degradation, while P3 revealed almost similar protein levels as NPC1^WT^ (Figure 1C).

**Figure 2:**
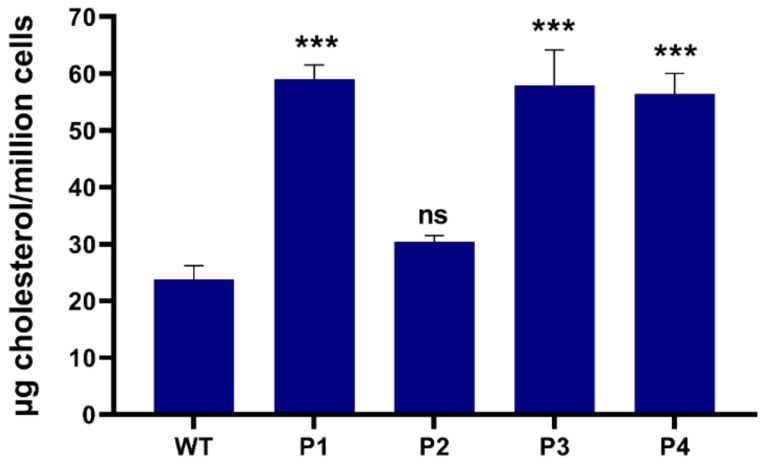
Cellular cholesterol contents in patients derived fibroblasts. After cholesterol extraction, total cholesterol levels measurement was performed with HPLC in fibroblasts derived from patient 1 (P1), patient 2 (P2), patient 3 (P3), patient 4 (P4) and wild-type fibroblasts (WT). Ordinary one-way ANOVA, ns *p* > 0.05 and *** *p* < 0.001 versus WT, S.E.M., and *n* = 3.

We subsequently assessed the impact of NPC1^MUT^ on cholesterol accumulation, a prominent characteristic of NPC diseases. For this purpose, total cellular cholesterol levels were measured by HPLC. Fibroblasts derived from patients 1, 3, and 4, showed a significantly higher level of cholesterol (>2 fold) compared to control WT fibroblasts. In contrast, the cholesterol levels in fibroblasts derived from patient 2 remained within the normal range (Figure 2).

### 2.2. Mutation-Dependent Rescue of NPC1 Trafficking in NPC Patients’ Fibroblasts

As indicated above (Section 2.1), the NPC1 protein exhibits different trafficking phenotypes depending on the mutation and its localization. These variations not only contribute to the clinical phenotype severity in NPC patients, but also influence the patient’s response to approved therapies, such as miglustat or cyclodextrin, thus proposing personalized therapies and drug combinations. Here, we examined the impact of RCME on the NPC1 trafficking and cholesterol levels in the four cell lines and the WT fibroblasts. RCME, enriched in polyphenols and flavonoids, previously demonstrated its ability to restore the balance of cholesterol- and sphingolipids-enriched lipid rafts in intestinal epithelial cells [12]. Building on this, we hypothesized that RCME may also impact the lipid rafts imbalance that is elicited by NPC1 mutations [10] by reducing the cholesterol levels.

**Figure 3:**
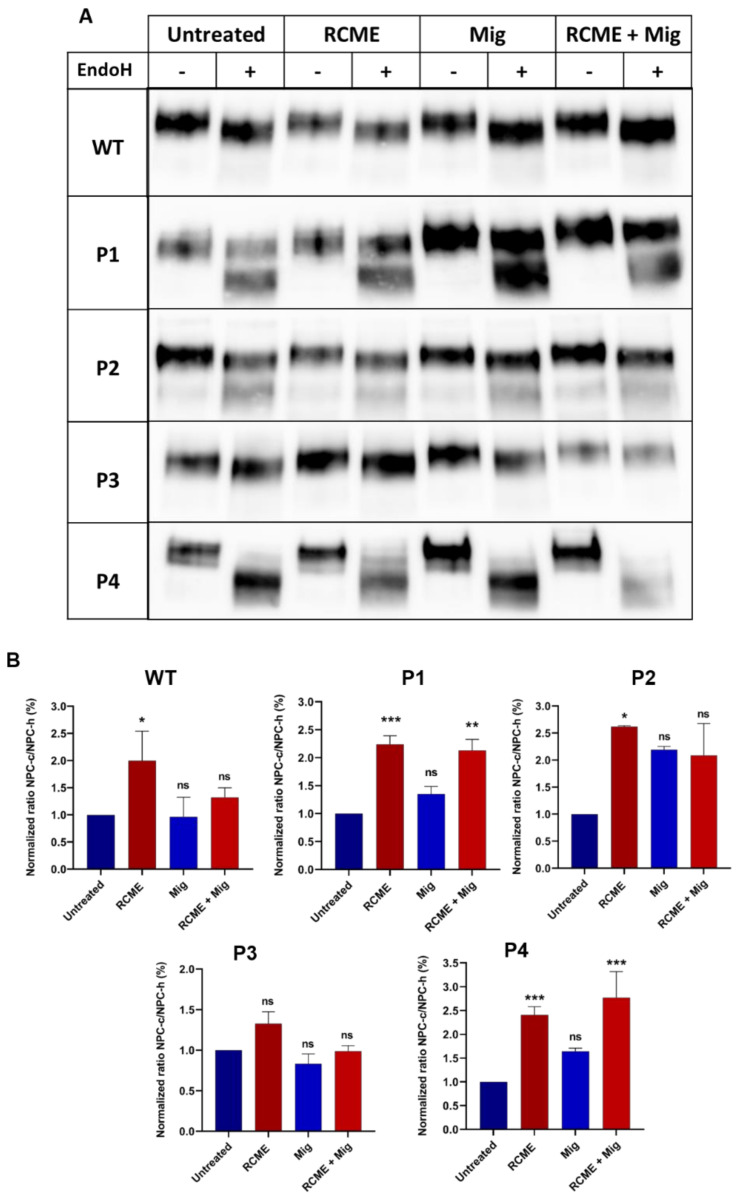
*Rosa canina* methanol extract (RCME) treatment enhanced the trafficking of NPC1 protein between the ER and the Golgi in a mutation-dependent manner. The fibroblasts derived from a healthy donor (WT), patient 1 (P1), patient 2 (P2), patient 3 (P3), and patient 4 (P4) were treated for 24 h with RCME, miglustat (Mig) or RCME + Mig. The cellular lysates were immunoprecipitated with anti-NPC1 and further treated or not treated with endo H. The immunoprecipitants were subjected to SDS-PAGE and Western blot for NPC1 analysis (**A**). The ratio of the complex (NPC-c) versus mannose-rich (NPC-h) forms of the NPC1 protein were depicted and the values were normalized to the untreated group (**B**). Ordinary one-way ANOVA, ns *p* > 0.05, * *p* < 0.05, ** *p* < 0.01 and *** *p* < 0.001 versus untreated group, S.E.M., and *n* = 3.

Fibroblasts from different patients or healthy donors were treated with RCME, miglustat, or a combination of both for 24 h. Endo H treatment was employed to monitor the trafficking profiles based on the proportions of the immature mannose-rich (NPC-h) and complex glycosylated (NPC-c) NPC1 forms. The results reveal a significant 2.5-fold increase in the proportions of the mature complex glycosylated form of NPC1 upon treatment of the cells from patients P1, P2, and P4 with RCME (Figure 3A,B). Miglustat treatment showed a slight, non-significant improvement in NPC1 trafficking and glycosylation. Notably, the trafficking pattern of NPC1 in P3 was maintained upon RCME treatment (Figure 3A,B). Miglustat, when administered alone, did not affect the trafficking profiles of NPC1, which were essentially similar to those in the non-treated cells. However, in combination with RCME, a significant effect was observed in P4. This genotype-specific response supports the view that personalized approaches are necessary for NPC treatment [10], as disease symptoms and therapeutic responses can vary significantly between individuals (Figure 3A,B).

While RCME treatment was able to counteract the effect of certain mutations and restore the trafficking of NPC1 protein, we sought to determine whether RCME rectified the NPC1 trafficking in the patients’ fibroblasts to achieve a wild-type-like state. RCME successfully restored the trafficking pattern of NPC1 in P1 and P2 to a state similar to NPC1^WT^, and improved the trafficking of NPC1 in P4, although it did not acquire wild-type-like levels.

### 2.3. RCME Reduces Cholesterol Accumulation More Efficiently than Miglustat

**Figure 4:**
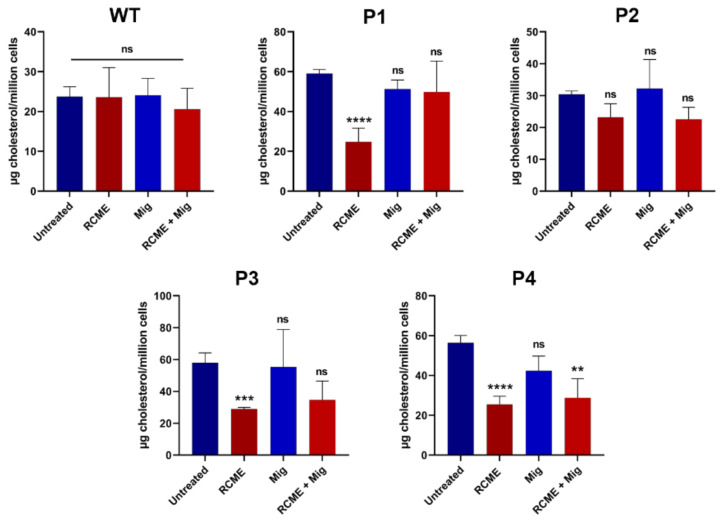
*Rosa canina* methanol extract (RCME) treatment inhibited the cholesterol accumulation in NPC1 patients. The fibroblasts derived from a healthy donor (WT), patient 1 (P1), patient 2 (P2), patient 3 (P3), and patient 4 (P4), were treated for 24 h with RCME, miglustat (Mig) or RCME + Mig. After cholesterol extraction, the measurement was performed with HPLC. Ordinary one-way ANOVA, ns *p* > 0.05, ** *p* < 0.01, *** *p* < 0.001, and **** *p* < 0.0005 versus untreated group, S.E.M., and *n* = 3.

Current therapeutic options for NPC patients include miglustat, which targets glycosphingolipids that are also elevated in NPC patients, and cyclodextrin, known for reducing cholesterol levels. However, patients’ responses to these drugs vary significantly, rendering the search for other therapies necessary. In addition to its action on the folding and trafficking of NPC1 mutants, as shown above, RCME was shown to restore cholesterol- and sphingolipids-enriched lipid rafts in DSS-treated Caco-2 cells [12]. We therefore hypothesized a potential dual function of RCME in restoring NPC1 trafficking and in modulating cholesterol levels. The fibroblasts were hence treated with RCME or miglustat and a combined treatment to investigate a potential reducing effect on cholesterol accumulation caused by mutations in the NPC1 gene. Concomitantly, to determine if RCME or miglustat have an overall effect on total cholesterol level, the WT fibroblasts were also treated. As depicted in Figure 4, none of the treatment conditions exerted an influence on the cholesterol level in WT fibroblasts, which remained comparable to the untreated fibroblasts. The same effect was noted in P2, where the cholesterol level remained constant under the different treatment conditions (Figure 4).

The fibroblasts derived from the patients P1, P3, and P4 exhibited significantly higher cholesterol levels in comparison to the WT fibroblasts (Figure 2). Following treatment with 100 µg/mL RCME, these patient-derived fibroblasts displayed an average cholesterol level of 26.4 µg cholesterol/million cells, representing a notable 50% reduction compared to the untreated cells, which exhibited 57.8 µg cholesterol/million cells. Miglustat treatment resulted in only a modest reduction in cholesterol levels likely due to the short treatment duration (24 h). On the other hand, in a study by Brogden et al. [11], miglustat demonstrated a significant cholesterol-lowering effect after 72 h of treatment. The combined treatment of RCME and miglustat reduced cholesterol levels by approximately 50% to 28.74 µg cholesterol/million cells (*p* < 0.01) in P4-derived cells (Figure 4).

Cholesterol analysis revealed that RCME inhibited cholesterol accumulation in the three patients (P1, P2, and P3) that exhibited higher cholesterol levels than the healthy donor. The findings indicate that, following RCME treatment, cholesterol levels in the patient-derived fibroblasts are similar to the levels in the WT cells. This observation prompted the question: is the effect of RCME on cholesterol accumulation due to its effect on the NPC1 protein, the direct modulation of cholesterol synthesis, or a combination of both factors?

**Figure 5:**
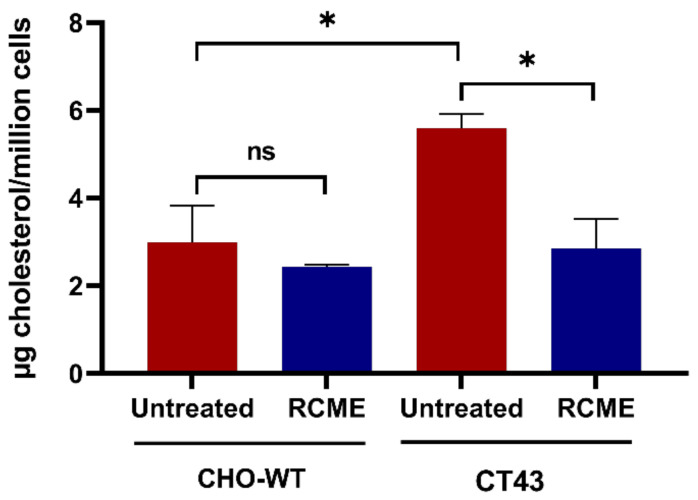
Cholesterol level was restored in NPC1-knocked out CHO (CT43) cells upon treatment with *Rosa canina* methanol extract (RCME). The wild-type Chinese hamster ovary (CHO-WT) cells and NPC1-knocked out CHO (CT43) cells were treated for 24 h with RCME. After cholesterol extraction, the measurement was performed with HPLC. Ordinary one-way ANOVA, ns *p* > 0.05, * *p* < 0.05 versus untreated group, S.E.M., and *n* = 3.

To further investigate the aforementioned hypothesis, we employed wild-type Chinese hamster ovary cells (CHO-WT) and NPC1-knocked out CHO cells (CT43). These cells were treated with RCME for 24 h and then the total cholesterol level was determined in comparison to untreated cells. The difference in the cholesterol amount between the CHO-WT and CT43 is depicted in Figure 5, where the CT43 cells revealed a significantly higher cholesterol level compared to the CHO-WT. Notably, within CHO-WT, the cholesterol level remained unchanged upon RCME treatment. However, RCME induced a significant 50% decrease in total cholesterol in CT43 cells. Remarkably, the cholesterol content in RCME-treated CT43 was reduced to an equal level as in the CHO-WT, 2.99 to 2.85 µg cholesterol/million cells, respectively.

### 2.4. The Effect of Rutin and Quercitrin on Cholesterol Reduction

Rutin and quercitrin are the major polyphenolic components of RCME [14] and are likely to trigger the observed positive effects of RCME on reducing cholesterol levels in patients’ fibroblasts. We therefore investigated the role of each of these components separately and utilized filipin staining, a standard diagnostic measure in NPC fibroblasts by which intracellular cholesterol accumulation can be visualized. The cells were treated with 10 mM of either rutin or quercitrin for 24 h.

**Figure 6:**
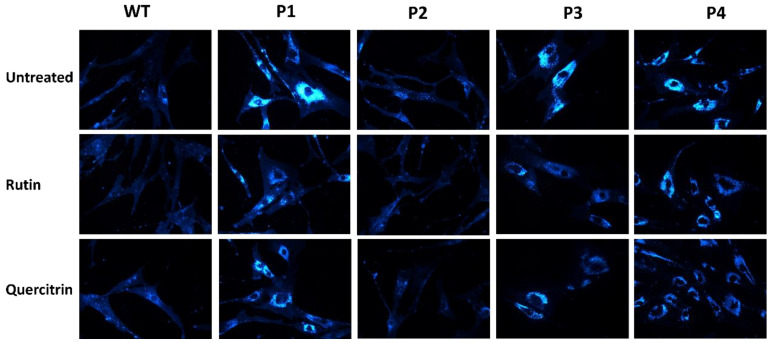
Filipin staining revealed reduction in lysosomal cholesterol accumulation after treatment with rutin (10 mM) or quercitrin (10 mM) in fibroblasts derived from healthy donor (WT) or NPC patients, patient 1 (P1), patient 2 (P2), patient 3 (P3), and patient 4 (P4). Magnification 60×.

As shown in Figure 6, the cells from P1, P3, and P4 exhibited high filipin staining levels as compared to the WT cells, consistent with their elevated cholesterol obtained from HPLC measurements (Figure 2). Upon treatment with rutin or quercitrin, a clear reduction in the staining intensity was observed, compatible with reduced cholesterol accumulation.

**Figure 7:**
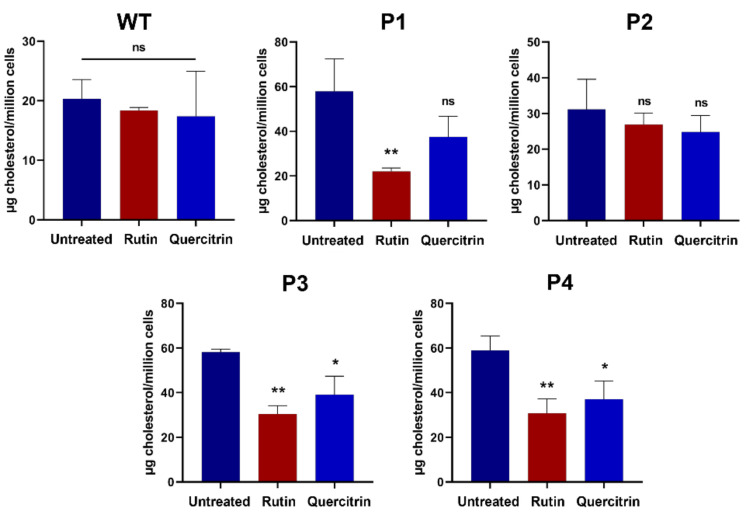
Rutin and quercitrin inhibited the cholesterol accumulation in NPC1 patients. The fibroblasts derived from a healthy donor (WT), patient 1 (P1), patient 2 (P2), patient 3 (P3), and patient 4 (P4) were treated for 24 h with rutin (10 mM) or quercitrin (10 mM). After cholesterol extraction, the measurement was performed with HPLC. Ordinary one-way ANOVA, ns *p* > 0.05, * *p* < 0.05 and ** *p* < 0.01 versus untreated group, S.E.M., and *n* = 3.

These qualitative observations were further corroborated quantitatively by measurement of cellular cholesterol levels using HPLC. The fibroblasts derived from P1, P3, and P4 demonstrated a significant decrease in cholesterol levels upon treatment with rutin or quercitrin (Figure 7).

**Figure 8:**
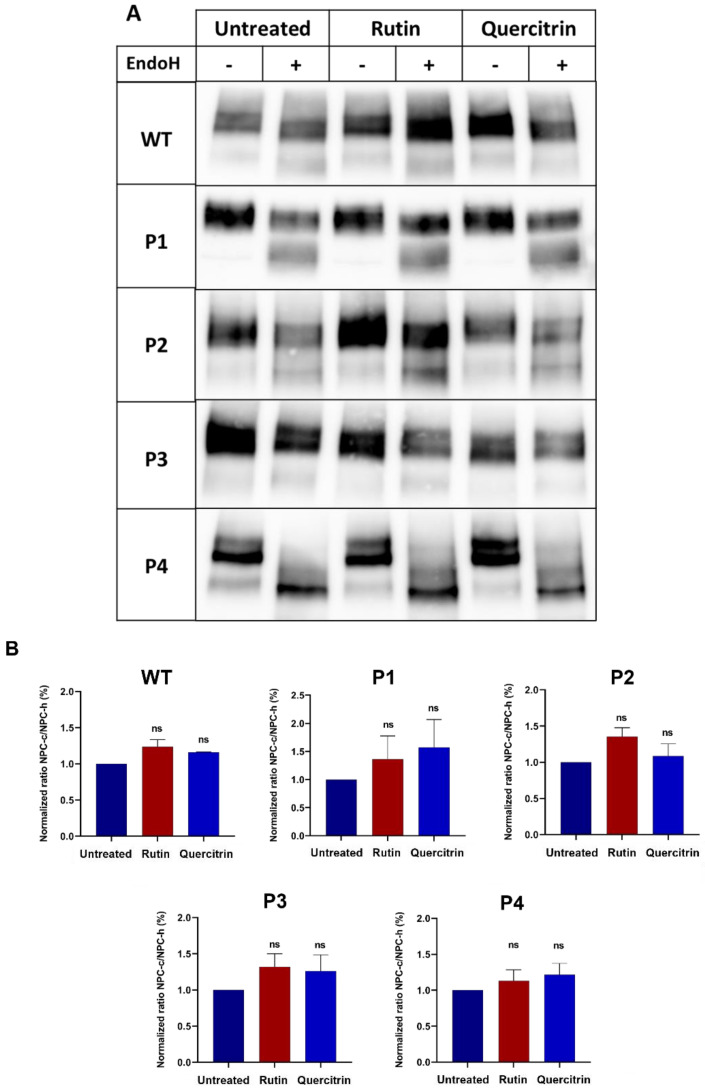
Effect of rutin and quercitrin on the trafficking of NPC1 protein between the ER and the Golgi. The fibroblasts derived from a healthy donor (WT), patient 1 (P1), patient 2 (P2), patient 3 (P3), and patient 4 (P4) were treated for 24 h with rutin (10 mM) or quercitrin (10 mM). The cellular lysates were immunoprecipitated with anti-NPC1 and further treated or not treated with endo H. The immunoprecipitates were subjected to SDS-PAGE for NPC1 analysis (**A**). The ratio of the complex (NPC-c) versus mannose-rich (NPC-h) forms of the NPC1 protein were depicted and the values were normalized to the untreated group (**B**). Ordinary one-way ANOVA, ns *p* > 0.05 versus untreated group, S.E.M., and *n* = 3.

We addressed a potential role of these two polyphenols at the ER level by positively impacting the trafficking pattern of NPC1 as is shown above for the crude RCME (see Figure 3). The results reveal that upon rutin or quercitrin treatment, the ratio NPC-c/NPC-h was not affected in either WT or patients’ fibroblasts (Figure 8), suggesting that the role of these two components is either at the level of cholesterol synthesis or in the Golgi.

## 3. Discussion

The heterogeneity and severity of symptoms in the neurodegenerative NPC disease are linked to the genetic makeup of the patients that subsequently impact the responsiveness to currently available therapies. Since NPC is a protein trafficking and folding disease that is elicited by mutations that affect its transport along the secretory pathway to varying extents, improving and enhancing the trafficking of NPC1 mutants to the lysosomes should be considered when designing new or combining available therapies. Currently available therapies target exclusively lipid membrane constituents with variable responsiveness and symptoms improvements. Miglustat, as already mentioned, is an iminosugar that is currently approved for treatment of NPC in European countries. It acts by reducing the accumulation of gangliosides in the brain [19] and the levels of cholesterol via the inhibition of sphingolipid synthesis, thus restoring membrane lipid homeostasis [20]. Hydroxypropyl-β-cyclodextrin (HPB-CD), another drug approved by the FDA for NPC, modifies cell membrane lipid composition by complexing with and extracting cholesterol and phospholipids from the lipid bilayers [21].

The observations in this study demonstrate that the polyphenolic extract RCME targets cholesterol and its overall cellular reduction as well as the trafficking pattern of NPC1 mutants. However, it is important to note that while rutin and quercitrin effectively reduced cholesterol levels, they did not significantly improve NPC1 trafficking. This differential effect on cholesterol without improving NPC1 trafficking underscores the importance of tailored therapeutic treatments, in view of the differential trafficking patterns of NPC1 mutants, which can be (i) completely blocked in the ER, (ii) transported normally, or (iii) at a low rate to the lysosomes. NPC1 proteins harboring the mutations p.I1061T or p.P887L in P1 are characterized by intracellular block in the ER [10], but together exhibit delayed trafficking of the resulting dimeric NPC1 protein [11]. On the contrary, NPC1 protein in fibroblasts of P4, who is homozygous for p.R1266Q, is blocked in the ER, while NPC1 in P2, homozygous to p.N1156S, and P3, heterozygous to V1165M, revealed trafficking patterns similar to that of wild-type NPC1. These apparent variabilities in the trafficking patterns and substantial reduction in the expression of the NPC1 protein translate into variations in cellular cholesterol levels in P1 and P4, which are highly elevated and compatible with delayed trafficking of NPC1 protein or its block in the ER. Surprisingly and in sharp contrast to the above, a direct association between the trafficking of NPC1 from the ER to the Golgi and cholesterol levels cannot be drawn when P3 is considered, since NPC1 in this specific case is normally trafficked, matures in the Golgi, and reveals additionally similar expression levels to wild-type NPC1. Here, an impaired binding of this NPC1 mutant to cholesterol in the lysosome could be responsible for the increased cellular cholesterol levels or targeting of NPC1, at least in part, to the cell surface rather than to the lysosome may have occurred. In silico modeling of several of these mutants revealed reduced docking of cholesterol onto the cholesterol binding domain, which may explain in part the lack of trafficking of cholesterol out of the lysosomes [22]. These observations with patients’ fibroblasts reveal once again how heterogeneity in the pathogenesis of NPC is strictly linked to the genotype and subsequently protein phenotype, necessitating therapeutic measures that target enhancement of protein trafficking as well as reduction in cholesterol levels. Neither miglustat nor ß-methyl cyclodextrin fulfill these two criteria, whereas RCME does, as revealed in the partial restoration for NPC1 trafficking, as well as the normalization of cholesterol levels upon RCME treatment.

Rutin and quercitrin, the major components of RCME, were found to significantly reduce cholesterol accumulation in NPC patient fibroblasts. This hypolipidemic effect is crucial because cholesterol accumulation is a hallmark of NPC disease. Moreover, the direct effect of RCME on the lipid concentration is corroborated by the restoration of cholesterol levels in NPC1-knockout CHO cells. RCME is a crude extract with a high flavonoid concentration level [14]. A subclass of flavonoids, the isoflavonoids, namely genistein, showed the ability to reduce cholesterol accumulation in NPC patient´s fibroblasts [23]. Previous reports show that lysosomal cholesterol accumulation suppresses the endosome–lysosome trafficking [24] and reduces the autophagolysosome formation [25]. Moreover, impaired autophagy in NPC is strongly associated with cholesterol accumulation [26]. Another important component is quercitrin, a glycosylated form of quercetin and the most abundant compound in the RCME. This component is capable of inhibiting the increase in body fat, liver weight, and the development of hepatic steatosis in ovariectomized mice, and is able to decrease the levels of serum lipid metabolites, including triglycerides, total cholesterol, and LDL-cholesterol [27]. These findings support the effect of quercitrin in improving dysregulated lipid metabolism. Several studies report also that rutin, another flavonoid found in RCME, decreases serum levels of triglyceride, LDL, and very low-density lipoprotein in experimental models of diabetes [28,29].

RCME utilized in this study enhanced the trafficking of NPC1 substantially, even to levels close to those of NPC1^WT^. However, the treatment with rutin or quercitrin did not affect NPC1 trafficking substantially, suggesting that several components in the RCME are likely implicated in this overall effect rather than one individual bioactive compound. The combined protective effects of bioactive compounds from plant extracts is well-documented in the literature [30]. Restoration of protein trafficking with RCME was reported in a previous study, in a Caco-2 cell model of DSS-induced colitis, in which the impaired trafficking and polarized sorting of sucrase-isomaltase and dipeptidyl-peptidase were restored with RCME treatment [12]. Previous studies highlighted the impact of flavonoids on protein stability and folding and acting as chemical chaperones in preventing protein misfolding and aggregation [31,32]. Since acquisition of correct protein folding and transport competence occurs initially in the ER along the constitutive secretory pathway, we conclude that the RCME primary site of action is the ER. Except for NPC1 in P3, the other NPC1 variants are fully or partially blocked intracellularly in the ER and are expected to trigger ER stress and unfolded protein response (UPR), as was shown for an NPC1 mutant harboring a silent variant p.Val562Val that leads to the skipping of exon 11 and premature stop codon [33]. Accordingly, targeting components implicated in ER stress may constitute a therapeutic option in the treatment of NPC. Previous data show that RCME is capable of normalizing the levels of ER stress proteins in an ER stress-induced cellular model [12]. In contrast, miglustat treatment did not positively impact protein trafficking.

The variation in the effectiveness of *Rosa canina* methanolic extract across different NPC1 mutants highlights the heterogeneity in disease phenotypes. Mutations such as NPC1^I1061T^, which cause protein misfolding, may be less responsive to treatments that target trafficking defects, while mutations such as NPC1^R1266Q^ might show improved responses due to their specific functional impairments. This variability underscores the potential limitations of applying a single therapeutic approach across all patients with NPC1 mutations and emphasizes the importance of personalized medicine in NPC treatment. Further studies are needed to explore how *Rosa canina* could be integrated into personalized therapeutic regimens, potentially in combination with other treatments targeting specific genetic mutations.

## 4. Materials and Methods

### 4.1. Cell Culture and Treatments with RCME and Miglustat

Skin-derived fibroblasts from NPC patients, carrying homozygous, heterozygous, or compound heterozygous NPC1 mutations [34,35], were obtained from Prof. Carlo Dionisi-Vici, Unit of Metabolic Disease at the Bambino Gesù Children’s Hospital in Rome, Italy. The study was performed within the AIFA trial with protocol number FARM59T23W and therefore consent was given to use these fibroblasts. Another source was the Clinic for Pediatric Kidney, Liver and Metabolic Diseases of the Medical School Hannover, Hannover, Germany. Patients gave informed consent for using the cells for scientific purposes. The ethical review board of Hannover Medical School granted a positive vote (EC Nr.5176) for studying metabolism in human fibroblasts [11]. The wild-type (WT) fibroblast cell line was used as a control. The used cell lines and their mutations in the coding DNA and amino acid sequence are listed in Table 1. The fibroblasts were cultured to confluence in low glucose (1 g/L) Dulbecco’s Modified Eagle’s Medium (DMEM; Sigma, Darmstadt, Germany), 10% fetal calf serum (FCS, Gibco BRL, Grand Island, NY, USA) (*v*/*v*), 100 U/mL penicillin, and 100 μg/mL streptomycin (Sigma, Darmstadt, Germany) at 37 °C in 5% CO_2_. Wild-type Chinese hamster ovary (CHO-WT) cells and NPC1-knockout CHO (CT43) cells were maintained in RPMI 1640 medium (Sigma, Darmstadt, Germany), 10% fetal calf serum (FCS) (*v*/*v*), 100 U/mL penicillin, and 100 μg/mL streptomycin at 37 °C in 5% CO_2_.

RCME was prepared from *Rosa canina* L., as described in Wanes et al. [14], and was dissolved in DMSO prior to use.

The cells were treated with either 100 µg/mL RCME, 100 µM *N*-butyl deoxynojirimycin (miglustat/Actelion, Allschwil, Switzerland), 10 mM rutin (Merck, Darmstadt, Germany), or 10 mM quercitrin (Merck, Darmstadt, Germany) for 24 h and compared to control, untreated fibroblasts.

### 4.2. Endoglycosidase H Treatment

To analyze the biosynthetic forms of NPC1, the fibroblasts were lysed in a 25 mM Tris buffer (pH 7.4) containing 0.5% SDS and protease inhibitors. The lysate was kept rotating for one hour at 4 °C and was then centrifuged for 20 min at 17,000× *g* to remove cell debris. The cellular lysates were immunoprecipitated with an anti-NPC1 antibody. The immunoprecipitates were first denatured using 5% SDS and 10% 2-mercaptoethanol for 90 min at 37 °C with continuous shaking, then these were treated with G5 buffer (0.5 M sodium citrate, pH 5.5) and 0.5 µL (5 U/mL) endoglycosidase H (endo H, Roche Diagnostics, Mannheim, Germany) for two hours on a shaker at 37 °C. During this process, asparagine-linked hybrid or high-mannose oligosaccharides in the ER are cleaved by endo H.

### 4.3. SDS-PAGE, Western Blotting

The immunoprecipitates were resolved under reducing conditions (10 mM dithiothreitol) by SDS-PAGE on 8% polyacrylamide gels according to Laemmli [36]. After gel electrophoresis, the protein bands were transblotted on PVDF membranes which were blocked in skimmed milk (5%) and PBS-Tween (0.1%) for one hour at room temperature. Immunoblotting was performed with an anti-NPC1 antibody (Sigma-Aldrich, St. Louis, MO, USA), anti-Flotillin 2 (Santa Cruz Biotechnology, Dallas, TX, USA), anti-Actin (Santa Cruz Biotechnology, TX, USA), and HRP-conjugated secondary anti-mouse or anti-rabbit antibodies (Thermo Fisher, Schwerte, Germany). The protein bands were visualized using the ChemiDoc system and enhanced chemiluminescence (ECL) with the SuperSignal™ West Femto Maximum Sensitivity Substrate using the ChemiDoc MP™ Touch Imaging System (Bio-Rad, Munich, Germany). Quantification of the protein band intensities was performed with Image Lab software 6.1.

### 4.4. Filipin Staining

Healthy or patient-derived fibroblasts were pre-seeded on coverslips. In the 24 h post treatment, the cells were washed twice with PBS. The cells were subsequently fixed with 4% paraformaldehyde (PFA) for 30 min and then quenched with 50 mM ammonium chloride (NH_4_Cl) for another 30 min. The cells were then stained with filipin reagent (dilution 1:25, Sigma-Aldrich, St. Louis, MO, USA) for two hours in the dark. All steps were performed at room temperature. A Leica DM IRB fluorescent microscope was used to visualize the images with a 60× objective.

### 4.5. Cholesterol Analysis

The fibroblasts were washed twice with PBS after 24 h of incubation with miglustat, RCME or the combination treatment. Using trypsin-EDTA, the cells were detached, resuspended in media, transferred to a falcon tube, and centrifuged at 4°C for 5 min at 8000× *g*. The media was discarded, and the cells were washed once with PBS. Following this, 1 mL of PBS was added to the cell pellet which was placed on ice. After counting the cells using a Neubauer counting chamber, 0.5–1.5 million cells were aliquoted per sample and homogenized using a 26 G needle (20 times). The lipids were then isolated using a chloroform/methanol extraction protocol as described by Bligh and Dyer [37], with minor adjustments according to Brogden et al. [11].

For the lipid analysis, 250 µL of a chloroform/methanol solution were added to each lipid sample. After 5 min, the sample was transferred into a brown glass tube suitable for HPLC measurements. Cholesterol levels were measured using a Hitachi Chromaster HPLC (Hitachi High-Tech Corporation, Tokyo, Japan) system equipped with a Chromolith^®^ HighResolution RP-18 endcapped column (100 × 4.6 mm) along with a 5 × 4.6 mm guard cartridge. Methanol was utilized as the mobile phase, with a constant flow rate of 1 mL/min and operating pressure of 22 bar, using an isocratic elution method. Detection was performed using a UV detector (Hitachi High-Tech Corporation, Tokyo, Japan) set to 202 nm. Cholesterol concentrations in each sample were determined by comparison with an external standard, prepared with concentrations ranging from 0.05 to 2 mg/mL, and the results are expressed as micrograms of cholesterol per million cells. The method demonstrated linearity within this standard range. Measurements below the minimum detection limit were excluded from the analysis.

### 4.6. Statistical Analysis

Statistical analyses were performed using GraphPad Prism 8.0.1 (244) software (GraphPad Software, San Diego, CA, USA). Data are shown as mean ± standard deviation of at least three independent experiments. Two-way ANOVA followed by Tukey’s multiple comparisons test for the lipid analysis and Šídák’s multiple comparisons test for the trafficking experiments were used for the comparison between the different groups. *p*-values less than 0.05 were considered statistically significant.

## 5. Conclusions

The findings from our study reveal a promising potential for *Rosa canina* L. methanol extract (RCME) to mitigate protein trafficking aberrations and cholesterol accumulation in different phenotypes of Niemann–Pick C1 (NPC1). The comprehensive analysis of treatment outcomes sheds light on the intricate interplay between RCME, NPC1 protein function, and cholesterol homeostasis.

## Figures and Tables

**Table 1:** Characteristics of fibroblast cell lines from the control and patients with the respective mutations, genotypes, and protein-trafficking phenotypes.

Patient (P)	Mutation		Inheritance Pattern (Genotype)
	cDNA	Protein	
Patient 1	c.3182T>C c.3337C>T	p.I1061T p.P887L	Compound heterozygous
Patient 2	c.3467A>G	p.N1156S	Heterozygous
Patient 3	c.3493G>A c.3591+121C>T	p.V1165M p.0	Compound heterozygous
Patient 4	c.3797G>A	p.R1266Q	Homozygous

## Data Availability

All generated data is included in the article.
